# Emergence of chicken infection with novel reassortant H3N8 avian influenza viruses genetically close to human H3N8 isolate, China

**DOI:** 10.1080/22221751.2022.2128437

**Published:** 2022-10-26

**Authors:** Zhimin Wan, Wenjie Jiang, Jianxi Gong, Zhehong Zhao, Ting Tang, Yafeng Li, Jianjun Zhang, Quan Xie, Tuofan Li, Hongxia Shao, Jinhua Liu, Aijian Qin, Jianqiang Ye

**Affiliations:** a Key Laboratory of Jiangsu Preventive Veterinary Medicine, Key Laboratory for Avian Preventive Medicine, Ministry of Education, College of Veterinary Medicine, Yangzhou University, Yangzhou, People’s Republic of China; b Institute of Agricultural Science and Technology Development, Yangzhou University, Yangzhou, People’s Republic of China; c Jiangsu Co-innovation Center for Prevention and Control of Important Animal Infectious Diseases and Zoonoses, Yangzhou, People’s Republic of China; d Joint International Research Laboratory of Agriculture and Agri-Product Safety, the Ministry of Education of China, Yangzhou University, Yangzhou, People’s Republic of China; e Sinopharm Yangzhou VAC Biological Engineering Co. Ltd, Yangzhou, People’s Republic of China; f Key Laboratory for Prevention and Control of Avian Influenza and Other Major Poultry Diseases, Ministry of Agriculture and Rural Affairs, College of Veterinary Medicine, China Agricultural University, Beijing, People’s Republic of China

## Dear editor

Wild aquatic birds are the primary natural reservoir for most subtypes of avian influenza viruses (AIVs) [1]. These viruses often spill over to poultry and some can subsequently be transmitted to humans. In the past two decades, multiple subtypes of IAVs from chickens, including H5N1, H5N6, H7N9, H9N2, H10N3 and H10N8, etc., have caused human infections [2–5], with high mortality being recorded among the H5N1 and H7N9 cases. Chickens in the interspecies transmission of AIVs highlight the importance of continued surveillance of emerging AIVs that may pose a risk to public health. Here, we report the isolation and characterization of H3N8 viruses from domestic chickens with respiratory symptom, which are closely related to the index human H3N8 isolate [6].

**Figure 1.**
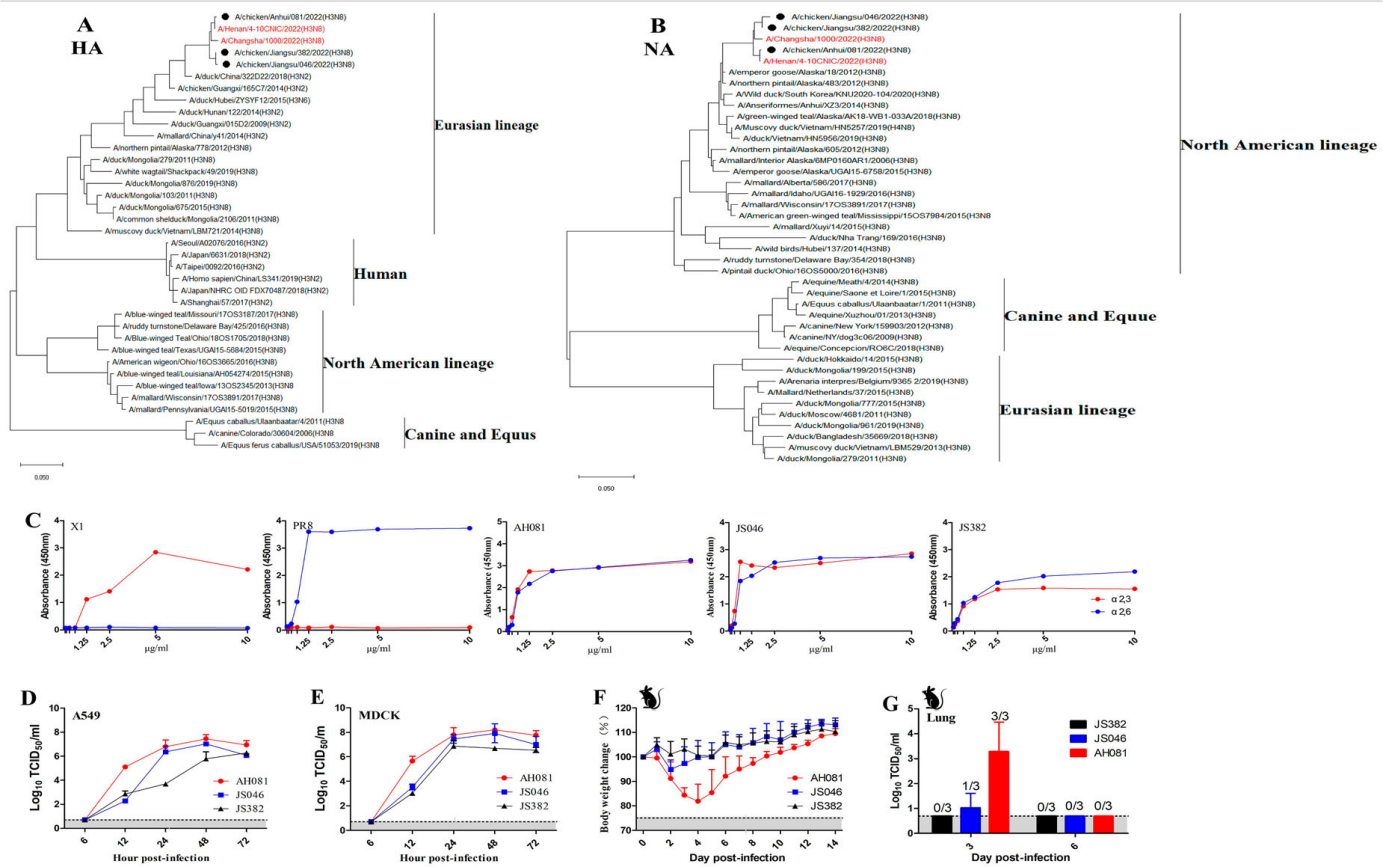
Phylogenetic trees of hemagglutinin (A) and neuraminidase (B) genes of the novel three H3N8 AIVs isolated from chickens in China, 2022. The trees were generated by maximum likelihood with the MAGE X software. The viruses from this study were labeled in black circles, the H3N8 AIVs that caused human infections in China were marked in red. Scale bars indicate branch length based on number of nucleotide substitutions per site. (C) Receptor-binding properties of the H3N8 viruses. The receptor binding of the H3N8 viruses was determined using various concentrations of sialic acid conjugated to biotinylated sialylglycopolymers (3′SLN and 6′SLN) via direct solid-phase binding assays, A/Chicken/Jiangsu/X1/2004(H9N2) (X1) and A/PuertoRico/8/1934(H1N1) (PR8) were selected as α-2,3 receptor and α-2,6 receptor controls, respectively. Growth kinetics of the H3N8 viruses in MDCK and A549 cells. MDCK and A549 cells were infected with each virus at an MOI of 0.001 (D and E). Supernatant samples were collected at 6, 12, 24, 48 and 72 hpi, and viral titers were measured in MDCK and A549 cells, respectively. Pathogenicity of the three H3N8 viruses in mice. Five-week-old BALB/c mice were infected intranasally with 10^5^ TCID_50_ units of each virus. Percentage of bodyweight change of mice infected with each virus (F). The viral titers of the lungs of the infected mice collected at 3 and 6 dpi were measured in MDCK cells (G).

During the winter season of 2021–2022, a respiratory disease occurred in 30–40 days old domestic chickens from several poultry farms in eastern China. Three H3N8 AIVs were isolated from the tracheal and lung samples from sick chickens and designated as A/chicken/Anhui/081/2022(H3N8) (AH081), A/chicken/Jiangsu/046 /2022(H3N8) (JS046) and A/chicken/Jiangsu/382/2022(H3N8) (JS382), respectively. To understand the origins of these H3N8 isolates, we sequenced their genomes and performed phylogenetic analysis. Phylogenetic trees were constructed by using the maximum likelihood in the software of MAGE X. The HA genes of these viruses were of Eurasian H3 lineage, which has been circulating in ducks since 2008 (Figure 1(A)). Their NA genes were genetically associated with those from H3N8 circulating in wild birds in North America (Figure 1(B)). Both HA and NA genes shared a high identity with those from the H3N8 that caused the first case of human infection [7]. The internal genes were genetically close to the G57 genotype H9N2, which has become predominant in chickens since 2010 in China [8]. The internal gene constellation, in general, is very similar to that of the human H3N8 isolate, especially AH081, sharing 99.5–100% nucleic acid homology with A/Henan/4-10CNIC/2022(H3N8).

The deduced amino acid sequences showed the three H3N8 AIVs shared the same amino acid motif (PEKQTR/GLF) at the cleavage site, indicating they belonged to low pathogenic strains. Moreover, all three H3N8 viruses carried residues Q226 and G228 in HA, typical for influenza viruses that bind to α2,3 sialic acid (SA) receptors [9,10]. Notably, although mutations E627K and D701N in PB2 for enhanced pathogenesis of AIVs in mammals were not found [11], other mutations that increased polymerase activity in mammalian cell lines and/or the pathogenicity to mammalian species were present in these H3N8 viruses. These included I292V, A588V and V598I in PB2, D622G in PB1, K356R in PA, N30D, I43M, T215A in M1, and P42S in NS1 [12], which mutations are also present in human H3N8 strain of A/Henan/4-10CNIC/2022, except for the amino acid V at position 598 of PB2. Interestingly, NS1 from AH081 possessed a 7-amino acid insertion (residues 231–237), which has been reported to enhance the replication and inflammatory cytokine production in chickens [13]. The NS1 of isolates JS046 and JS382 have a 13-amino-acid deletion (residues 218–230), which deletion has become predominant in H9N2 viruses circulating in poultry in China [13].

To determine the receptor specificity of the H3N8 viruses, the binding of these viruses to two receptor analogies, i.e. Neu5Acα2-3Galb1-4GlcNAcb (3′SLN) representative of ɑ-2,3 SA receptors and Neu5Acα2-6Galb1-4GlcNAcb (6′SLN) representative of ɑ-2,6 SA receptors, were tested by ELISA [14]. As shown in Figure 1(C), all viruses bound to both ɑ-2,3 SA and ɑ-2,6 SA. To identify the phenotypic diversity of these viruses in mammalian cells, we examined their growth kinetics in Madin-Darby canine kidney (MDCK) and human pulmonary epithelial A549 cells. Cells were infected with each virus at multiplicity of infection (MOI) of 0.001. All viruses replicated efficiently in MDCK and A549 cells, reaching peak titers at 48 h post-infection (Figure 1(D,E)). AH081 replicated faster than the other two viruses, especially at 12 h post-infection, the replication efficiency of AH081 was more than 100-fold compared to the other viruses in both cell lines (Figure 1(D,E)).

To investigate the infection potential of these H3N8 viruses in mammals, 5-week-old BALB/c mice were inoculated intranasally with 10^5^ TCID_50_ units of each virus. Clinical signs of disease, body weight change and mortality of the infected mice were recorded daily. As shown in Figure 1(F), the mice infected with AH081 showed bodyweight loss of around 20% within the first 4 days post-infection (dpi) and then slowly recovered. Interestingly, no signs of disease and only minor bodyweight loss were observed in mice infected with JS046 and JS382 isolate viruses (Figure 1(F)). The greater pathogenicity of AH081 was also reflected by the higher viral titer detected in lungs collected at 3 dpi (Figure 1(G)). Conversely, lower viral titers were observed in lung from mice infected with JS046 group at 3 dpi, and no virus was detected in the lung from mice infected with JS382 group at 3 dpi (Figure 1(G)), which was consistent with the replication efficiency of these three viruses *in vitro* (Figure 1(D,E)). At 6 dpi, no virus was detected in lungs from all three groups of mice (Figure 1(G)).

## Discussion

Here we present a preliminary study isolating and characterizing reassortant H3N8 viruses from chickens, which are genetically close to an index human isolate. These H3N8 viruses likely resulted from reassortment among viruses from ducks, chickens and wild birds and possessed molecular markers indicative of potential zoonosis at the animal and human interface. These include their dual receptor specificity for both avian- and human-like receptors, the internal gene constellation from H9N2 viruses with the characteristic of almost all of the AIVs caused human infections in the past two decades [2–5], high genetic similarity in the receptor-interacting genes HA and NA from the first human H3N8 isolate. Moreover, these three H3N8 viruses replicated efficiently in mammalian cells and AH081 is pathogenic to mice. Considering these as well as its wide and yet expanding host range [15], H3N8 virus should be closely monitored and its genesis, pathogenesis, and interspecies transmissibility, etc., should be continuously investigated.

## Ethics statement

The animal study was performed in accordance with the institutional animal guidelines and approved by the Animal Care Committee at Yangzhou University, China.
